# Oscillating behavior of *Clostridium difficile* Min proteins in *Bacillus subtilis*

**DOI:** 10.1002/mbo3.337

**Published:** 2016-01-27

**Authors:** Jana Makroczyová, Ján Jamroškovič, Eva Krascsenitsová, Nad'a Labajová, Imrich Barák

**Affiliations:** ^1^ Institute of Molecular BiologySlovak Academy of SciencesBratislavaSlovakia

**Keywords:** *Bacillus subtilis*, bacterial cell division, *Clostridium difficile*, Min system oscillation, sporulation

## Abstract

In rod‐shaped bacteria, the proper placement of the division septum at the midcell relies, at least partially, on the proteins of the Min system as an inhibitor of cell division. The main principle of Min system function involves the formation of an inhibitor gradient along the cell axis; however, the establishment of this gradient differs between two well‐studied gram‐negative and gram‐positive bacteria. While in gram‐negative *Escherichia coli,* the Min system undergoes pole‐to‐pole oscillation, in gram‐positive *Bacillus subtilis*, proper spatial inhibition is achieved by the preferential attraction of the Min proteins to the cell poles. Nevertheless, when *E.coli* Min proteins are inserted into *B.subtilis* cells, they still oscillate, which negatively affects asymmetric septation during sporulation in this organism. Interestingly, homologs of both Min systems were found to be present in various combinations in the genomes of anaerobic and endospore‐forming *Clostridia*, including the pathogenic *Clostridium difficile*. Here, we have investigated the localization and behavior of *C.difficile* Min protein homologs and showed that MinDE proteins of *C.difficile* can oscillate when expressed together in *B.subtilis* cells. We have also investigated the effects of this oscillation on *B.subtilis* sporulation, and observed decreased sporulation efficiency in strains harboring the MinDE genes. Additionally, we have evaluated the effects of *C.difficile* Min protein expression on vegetative division in this heterologous host.

## Introduction

Gram‐positive *Bacillus subtilis* and gram‐negative *Escherichia coli* are the most common model organisms used for studying cell division in rod‐shaped bacteria. Bacterial cell division is a strictly controlled, binary fission process leading to the formation of two equal daughter cells. FtsZ, a tubulin‐like protein, forms a structure termed the Z‐ring, which marks the position of the future division septum and serves as a scaffold for downstream division proteins. The placement of the division septum at the midcell site is very precise and the details of how this is achieved are still unknown. Two different mechanisms which have a negative effect on Z‐ring assembly have been described: nucleoid occlusion and the Min system (reviewed in Barák and Wilkinson 2007; Wu and Errington 2011; Rowlett and Margolin 2015). Recently, positive regulators of Z‐ring placement have been reported – the SsgAB system found in *Streptomyces coelicolor* (Willemse and van Wezel 2009), PomZ in *Myxococcus xanthus* (Treuner‐Lange et al. 2013), and MapZ in *Streptococcus pneumoniae* (Fleurie et al. 2014). The existence of a similar mechanism in *B.subtilis* has also been proposed (Monahan et al. 2014). It seems that Z‐ring placement is controlled differently by different bacterial species; many of the proteins involved in these systems are not highly conserved.

The Min system efficiently blocks unwanted polar septation during vegetative growth by creating a concentration gradient along the cell axis and hence protecting the polar sites from Z‐ring formation. The key component of the Min system is the MinC protein, which prevents Z‐ring formation by preventing FtsZ polymerization and by inhibiting interactions between FtsZ protofilaments (reviewed in Adams and Errington 2009). MinC is recruited to the cytoplasmic membrane, thereby triggering its inhibitory activity, by interacting with MinD, which binds reversibly to organized groups of anionic phospholipids within the membrane (Hu and Lutkenhaus 2001; Hu et al. 2002; Barák et al. 2008). The specific action and localization pattern of the MinCD complex at polar sites depends on an interaction with a third component of the Min system, termed the topological determinant. It is MinE in *E.coli* and DivIVA/MinJ in *B.subtilis*.

The behavior of *E.coli* Min proteins is based on a finely tuned interaction between MinE and MinD, and is highly dynamic. Upon binding of MinE to MinD, the ATPase activity of MinD is stimulated, resulting in the dissociation of the MinCD complex from the membrane and its reassociation at an adjacent site. This is manifested as rapid oscillation of all three proteins from one pole to the other, creating a bipolar MinC gradient and leaving only one place at the midcell site for FtsZ polymerization (Hu and Lutkenhaus 1999; Raskin and de Boer 1999a,b). *B.subtilis* does not encode a MinE homolog, on the other hand, and the polar localization of MinCD is achieved by an interaction with MinJ, which links the MinCD complex to the DivIVA protein (Bramkamp et al. 2008; Patrick and Kearns 2008). DivIVA stably localizes at the sites of septation based on its ability to bind to negatively curved membranes (Cha and Stewart 1997; Edwards and Errington 1997; Lenarcic et al. 2009; Eswaramoorthy et al. 2011), and it also persists at the cell poles. The preferential attraction of MinCD to the newly forming cell poles under the influence of MinJ/DivIVA blocks polar division in *B.subtilis*. This system is not entirely static: fast membrane dissociation and reassociation of *B.subtilis* MinD (MinD_Bs_), which retains its ATPase activity, has been observed (Barák et al. 2008), but MinD_Bs_ does not drive the rapid oscillation of MinC as *E.coli* MinD (MinD_Ec_) does in the presence of MinE_Ec_.

One of the possible paths of the *B.subtilis* cell cycle is sporulation, which begins with asymmetric cell division. During vegetative cell division, DivIVA helps position MinCD at the cell poles, but during sporulation it plays a role in the proper segregation of chromosomes (Thomaides et al. 2001) by attracting the RacA protein (Ben‐Yehuda et al. 2003). RacA recognizes the *oriC* region of elongated sister chromosomes and recruits these chromosomes to the cell poles (Thomaides et al. 2001). In addition, it was shown that DivIVA also interacts with SpoIIE, the most crucial protein for asymmetric cell division (Eswaramoorthy et al. 2014).

Min systems are not essential for cell viability, however, their absence has a clear effect on the cell phenotype. In *min* mutant strains, polar cell division produces mixtures of “mini” cells, which lack chromosomes, and extended rods containing multiple nucleoids (Adler et al. 1967; Reeve et al. 1973; de Boer et al. 1989). Furthermore, when *E.coli* MinD and MinE are introduced into *B.subtilis,* MinD_Ec_ oscillates just as it does in *E.coli* cells, and this oscillating system interferes with asymmetric septum formation during *B.subtilis* sporulation (Jamroškovič et al. 2012). Given the clear difference in the phospholipid composition of the *E.coli* and *B.subtilis* membranes (Kusters et al. 1991; López et al. 1998), this behavior was somewhat unexpected.

Many spore‐forming bacteria from the phylum *Firmicutes*, including the *Clostridia*, contain homologs from both MinCDE and MinCDJ/DivIVA systems. For example, the gram‐positive pathogenic spore‐former *Clostridium difficile* harbors homologs of MinC, MinD, MinE, and also DivIVA. Exactly which homologs are present varies according to the organism (Stragier 2002; Jamroškovič et al. 2012; this study) and it is not known whether they form a Min system which behaves as either of the two described. It is not even known whether all of these homologs are functional.

Because of the nature of *C.difficile* anaerobic lifestyle and its confined genetic toolbox, we have decided to address these questions by investigating the mechanism of action of the *C.difficile* Min proteins (Min_Cd_) in a heterologous *B.subtilis* host. We found that the Min proteins of *C.difficile* are functional in a heterologous host *B.subtilis* and can affect its vegetative division. We also found that the *C.difficile* MinD and MinE proteins exhibit oscillation, meaning that oscillating Min proteins are not confined only to gram‐negative bacteria. Oscillation of a YFP‐MinD_Cd_ fusion protein was observed in *B.subtilis* cells in the presence of MinE_Cd_. The same behavior can also be seen by combining the MinD and MinE proteins from *E.coli* and *C.difficile*, which opens interesting questions about the evolution of Min systems and the origins of gram‐positive and gram‐negative bacteria. Finally, we noted that the sporulation efficiency of those strains where oscillation was observed was diminished, indicating that either Min_Cd_ proteins or their oscillation interferes with the process of sporulation in *B.subtilis*.

## Experimental Procedures

### Culture conditions and bacterial strains

Strains were grown in LB (Luria‐Bertani) medium (Ausubel et al. 1987) or in DSM (Difco sporulation medium)(Schaeffer et al. 1965) at 37°C. DNA manipulations and *E.coli* transformations were performed according to Sambrook et al. (1989). The *B.subtilis* strains used in this work are derivatives of *B.subtilis* MO1099, and were prepared by transformation using plasmid or chromosomal DNA following the method of Harwood and Cutting (1990). All *B.subtilis* and *E.coli* strains used in this study are listed in Table 1 and details of their construction are in Table S1. When required, media were supplemented with ampicillin (100 *μ*g mLˉ^1^), spectinomycin (100 *μ*g mLˉ^1^), erythromycin (1 *μ*g mLˉ^1^), lincomycin (25 *μ*g mLˉ^1^), kanamycin (10 *μ*g mLˉ^1^ or 30 *μ*g mLˉ^1^), chloramphenicol (5 *μ*g mLˉ^1^), and tetracycline (5 *μ*g mLˉ^1^). To induce the expression of proteins under the control of the P_*xyl*_ and P_hyperspank_ promoters, the media were supplemented, respectively, with xylose at t_0_ to a final concentration of 0.02–0.5% (w/v) and IPTG (isopropyl β‐D‐1‐thiogalactopyranoside) to a final concentration of 0.1–0.5 mmol/L.

**Table 1:** Bacterial strains used in this study

Strain or plasmid	Genotype or description	Construction	Reference or origin
*B. subtilis*			
PY79	Prototrophic derivative of *B. subtilis* 168		Youngman et al. (1984)
MO649	*thrC::cat*		Guérout‐Fleury et al. (1996)
MO1099	*amyE::erm*		Guérout‐Fleury et al. (1996)
IB220	*spo0A::kan*		Schmeisser et al. (2000)
IB1056	*minD*_*Bs*_ *::cat, amyE::erm*		Barák et al. (2008)
IB1059	*minD*_*Bs*_ *::cat, amy::P*_*xyl*_ *‐gfp‐minD*_*Bs*_ *spc*		Pavlendová et al. (2010)
IB1111	*minD*_*Bs*_ *::cat, amyE::P*_*hyperspank*_ *‐yfp‐minD*_*Ec*_ *spc*		Pavlendová et al. (2010)
IB1112	*minD*_*Bs*_ *::cat, divIVA::tet amyE::P*_*hyperspank*_ *‐yfp‐minD*_*Ec*_ *spc*		Pavlendová et al. (2010)
IB1141	*minC*_*Bs*_ *::kan*		Pavlendová et al. (2010)
IB1242	*minD*_*Bs*_ *::cat, divIVA*_*Bs*_ *::tet, amyE::P*_*hyperspank*_ *‐yfp‐minD*_*Ec*_ *spc, thrC::P*_*xyl*_ *‐minE*_*Ec*_ *erm*		Jamroškovič et al. (2012)
IB1362	*minJ*_*Bs*_ *::kan*		Jamroškovič et al. (2012)
IB1371	*minCD*_*Bs*_ *::kan*		Jamroškovič et al. (2012)
IB1410	*thrC::P*_*xyl*_ *‐minE*_*cd*_ *erm*	MO649::pNP‐minE_Cd_	This study
IB1412	*minD*_*Bs*_ *::cat, amyE::P*_*hyperspank*_ *‐yfp‐minD*_*Ec*_ *spc, thr::P*_*xyl*_ *‐minE*_*Cd*_ *erm*	IB1111::chr DNA IB1410	This study
IB1413	*minD*_*Bs*_ *::cat, divIVA::tet, amyE::P*_*hyperspank*_ *‐yfp‐ minD*_*Ec*_ *spc, thr::P*_*xyl*_ *‐minE*_*Cd*_ *erm*	IB1112::chr DNA IB1410	This study
IB1415	*amyE::P*_*hyperspank*_ *‐yfp‐minD*_*cd*_ *spc*	MO1099::pED‐yfp‐minD_Cd_	This study
IB1416	*minD*_*Bs*_ *::cat, amyE::P*_*hyperspank*_ *‐yfp‐minD*_*Cd*_ *spc*	IB1056::chr DNA IB1415	This study
IB1553	*minJ*_*Bs*_ *::kan, amyE::P*_*hyperspank*_ *‐yfp‐minD*_*Cd*_ *spc*	IB1415::chr DNA IB1362	This study
IB1417	*amyE::P*_*hyperspank*_ *‐yfp‐minD*_*Cd*_ *spc, thrC::P*_*xyl*_ *‐minE*_*Cd*_ *erm*	IB1415::chr DNA IB1410	This study
IB1418	*minD*_*Bs*_ *::cat, amyE::P*_*hyperspank*_ *‐yfp‐minD*_*Cd*_ *spc, thrC::P*_*xyl*_ *‐minE*_*Cd*_ *erm*	IB1416::chr DNA IB1410	This study
IB1419	*minD*_*Bs*_ *::cat, minJ*_*Bs*_ *::kan, amyE::P*_*hyperspank*_ *‐yfp‐minD*_*Cd*_ *spc*	IB1416::chr DNA IB1362	This study
IB1545	*minD*_*Bs*_ *::cat, minJ*_*Bs*_ *::kan *	IB1056::chr DNA IB1362	This study
IB1546	*minD*_*Bs*_ *::cat, minJ*_*Bs*_ *::kan, amyE::P*_*hyperspank*_ *‐yfp‐minD*_*Cd*_ *spc, thrC::P*_*xyl*_ *‐ minE*_*Cd*_ *erm*	IB1419::chr DNA IB1410	This study
IB1549	*amyE::P*_*xyl*_ *‐minC*_*Cd*_ *spc*	MO1099::pSG‐minC_Cd_	This study
IB1550	*minC::kan, amyE::P*_*xyl*_ *‐minC*_*Cd*_ *spc*	IB1549::chr DNA IB1141	This study
IB1552	*amyE::P*_*xyl*_ *‐minE*_*Cd*_ *‐mgfp spc*	MO1099::pSG‐minE_Cd_‐mGFP	This study
IB1562	*minD*_*Bs*_ *::cat, minJ*_*Bs*_ *::kan, amyE::P*_*xyl*_ *‐gfp‐minD*_*Bs*_ *spc, thrC::P*_*xyl*_ *‐minE*_*Cd*_ *erm*	IB1059::chr DNA IB1362::chr DNA IB1410	This study
*E. coli*			
MM294	*F* ^*−*^ *endA1 hsdR17 (rk* ^*‐*^ *, mk) supE44 thi‐1 recA* ^*+*^		Meselson and Yuan (1968)
BTH101	*F* ^*−*^ *cya‐99 araD139 galE15 galK16 rpsL1(Str* ^*R*^ *) hsdR2 mcrA1 mcrB1*		Karimova et al.(1998)
*C. difficile*			
*C. difficile* 630	* *		kind gift from Prof. Neil Fairweather

### Construction of recombinant plasmids

Plasmids were constructed using standard methods and propagated in *E.coli* MM294; their construction is described in Table S1. Primers used in the study are listed in Table S2. All PCR fragments were amplified from the chromosomal DNA of the *C.difficile* 630 strain (kind gift from Prof. Neil Fairweather, Imperial College London). *C.difficile* has been recently renamed to *Peptoclostridium difficile* (Yutin and Galperin 2013), but we continue to use its traditional name here.

### Sporulation efficiency

The sporulation efficiency assay was performed as described in Harwood and Cutting (1990). Sporulation was induced by nutrient exhaustion in liquid DSM supplemented with 0.5 mmol/L IPTG, 0.5% xylose (w/v), and half the dose of the appropriate antibiotics at 37°C for 24 h after inoculation. Afterward, aliquots of the culture were serially diluted and plated on to LB plates before and after heat treatment (85°C, 15 min). Colonies formed from nontreated samples contain viable cells, those formed from heat‐treated samples contain cells that were able to sporulate. The sporulation efficiency was defined in terms of colony‐forming units (CFU) formed from nontreated samples (viable cells) and heat‐treated samples (spores), and was normalized against the sporulation efficiency of the originating strain. Each strain was assayed at least three times. The agar plates for photography were prepared by resuspending a single colony in 100 *μ*L of liquid DSM and applying 10 *μ*L of this suspension to DSM plates supplemented with 0.1 mmol/L IPTG and 0.02% xylose (w/v). These plates were sealed and incubated for 14 days at room temperature.

### Fluorescence microscopy and cell length determination

*Bacillus subtilis* strains were inoculated from a fresh overnight plate to an OD_600_ of 0.1 and grown as liquid cultures in DSM to the desired phase. Protein expression was induced at t_0_ by the addition of IPTG and/or xylose to a final concentration of 0.1–0.5 mmol/L and 0.02–0.3% (w/v), respectively. A small amount of culture was examined microscopically on freshly prepared poly‐L‐lysine‐treated slides or transferred to microscope slides covered with a thin layer of 1% agarose in LB. If necessary, cells were concentrated by centrifugation (3 min at 2300 *g*) and resuspended in a small volume of supernatant prior to microscopic examination. Cells and septal membranes were visualized by staining the cell cultures with FM 4–64 (Molecular Probes) at a concentration of 1 mg mL^−1^. YFP, GFP, and FM 4–64 fluorescence was observed using an Olympus BX63 microscope equipped with a Hamamatsu Orca‐R^2^ camera and analysed by Olympus Cell^P^ imaging software. The length of the *B.subtilis* cells was measured as described previously (Pavlendová et al. 2010). Briefly, *B.subtilis* cultures were grown as for fluorescence microscopy. Prior to examination, cultures were stained with FM 4–64. The cell length was taken to be the axis length from one cell pole to the other as measured using ImageJ (http://imagej.nih.gov/ij/). The average cell length was determined for at least 500 cells from each sample. Minicells were not included in the calculations of the average cell lengths.

### Bacterial two hybrid system

Fusions of MinD_Cd_ to the T18 and T25 fragments of adenylate cyclase were constructed for the bacterial adenylate cyclase two‐hybrid (BACTH) system (Karimova et al. 1998). The MinD_Cd_ sequence was PCR‐amplified using the respective primer pairs (Table S2) with the chromosomal DNA of *C.difficile* 630 as a template. These PCR fragments were then cloned into the BamHI/EcoRI sites of the pUT18, pUT18C, pKT25, and pKNT25 plasmids. Fusions of MinC_Bs_, MinD_Bs_, and MinJ_Bs_ in the BACTH system had been previously prepared (Jamroškovič et al. 2012). To test protein–protein interactions, the adenylate cyclase‐deficient *E.coli* BTH101 strain was cotransformed with various plasmid combinations and plated onto LB plates supplemented with X‐gal (40 *μ*g mL^−1^), IPTG (0.1 mmol/L), ampicillin (100 *μ*g mL^−1^), and kanamycin (30 *μ*g mL^−1^), and grown for 24–72 h at 30°C. Constructs were tested for autoinduction with the originating vectors containing only individual fragments of adenylate cyclase. The *β*‐galactosidase activity was measured as described by Miller (1972).

### Bioinformatic analysis

The NCBI's PSI‐BLAST program (Altschul et al. 1997) was used to search for homologs using the default threshold of 0.005 and to evaluate identity and similarity of homologous sequences. The sequences of the following strains were used in queries and alignments: *B.subtilis* (*Bacillus subtilis* PY79; taxid: 1415167), *C.difficile* (*Peptoclostridium difficile* 630; taxid: 272563), and *E. coli* (*E.coli* str. K‐12 substr. MG1655; taxid: 511145). Specific strains of clostridia were selected based on the availability of their whole genome sequence. The positions amphipatic helices were predicted using AmphipaSeek (Sapay et al. 2006). Multiple alignment of protein sequences was done using ClustalW plugin of CLC Sequence Viewer 7.6 software (CLC Bio, Cambridge, MA).

## Results

### *Clostridium difficile* Min proteins can influence *B.subtilis* cell division

**Figure 1:**
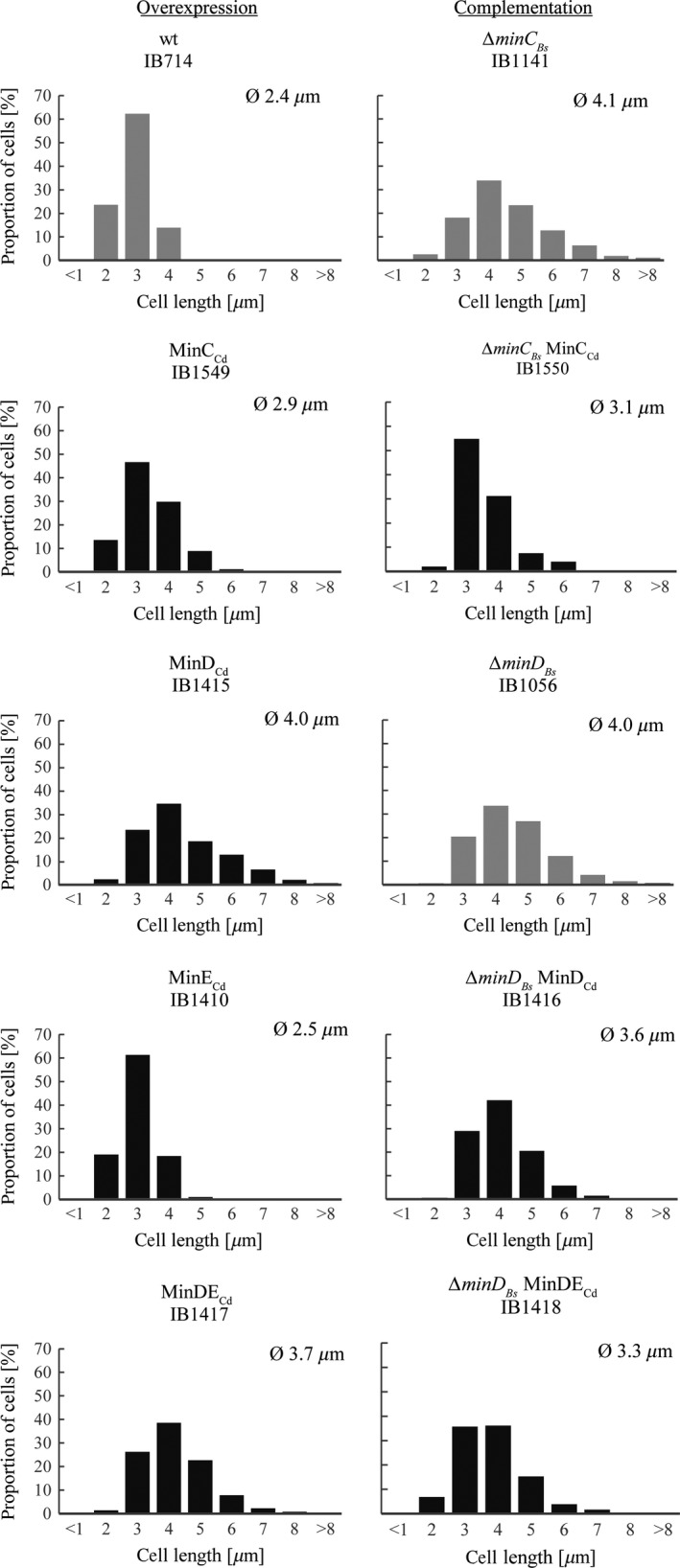
Cell length histograms. Left column: effects of overexpression of *C. difficile* Min proteins in wild‐type background. Expression was induced using 0.1 mM IPTG and/or 0.02% xylose, except for strain expressing MinC_C__d_ (IB1549), in which 0.3% xylose was used. Right column: complementation of Min_Bs_ proteins absence by Min_Cd_ proteins. Shown are induction conditions exhibiting the most notable complementation, that is 0.1 mM IPTG and/or 0.02% xylose except for strain *∆min* *D*_*B*__*s*_ MinDE_C__d_ (IB1418), in which higher concentrations were used (0.5 mM IPTG and 0.3% xylose). Parental strains are in gray. Summary of all measurements can be found in Table S3.

Our first question to address was whether the proteins of *C.difficile* are functional and could affect *B.subtilis* cell division. It was previously shown that higher expression of MinC_Bs_ and MinD_Bs_ in *B.subtilis* has a negative effect on bacterial cell division, resulting in elongation of the cells (Marston and Errington 1999). This effect was also observed when the *E.coli* MinC_Ec_ and MinD_Ec_ proteins were heterologously overexpressed in *B.subtilis* cells (Pavlendová et al. 2010). The average cell length of these elongated cells was 4 *μ*m. To examine the effect of the *C.difficile* Min proteins on *B.subtilis* cells, we placed the corresponding genes under the control of inducible promoters. The resulting strains are listed in Table 1. Measurements of cell length were performed with no inducer and with both low and high concentrations of inducer (low = 0.1 mmol/L IPTG and/or 0.02% xylose; high = 0.5 mmol/L IPTG and/or 0.3% xylose); the results are summarized in Table S3 and are illustrated in Figure 1. Additionally, the average cell length of the wild‐type strain (MO1099) was measured with and without the addition of xylose, to exclude its effect on cell division (not shown).

To explore the effect of *C.difficile* MinC (MinC_Cd_) on *B.subtilis* cell division, we placed the gene under the control of a xylose‐inducible promoter (P_*xyl*_) into an *amyE* locus. The cell length of the resulting IB1549 strain was measured without xylose and with of 0.3% xylose. This strain showed increased average length reaching 3 *μ*m and occurrence of cells longer than 4 *μ*m under both conditions up to 11% (Table S3). As the cell length also increased in the uninduced sample, it may be inferred that the cell division system of *B.subtilis* is so sensitive to MinC_Cd_ that even the low concentrations of it produced by a leaky P_*xyl*_ promoter (Vavrová et al. 2010) are enough to cause cell elongation. Leaky expression affected cell length in a previous Min system study as well (Pavlendová et al. 2010).

We also investigated the ability of MinD_Cd_ to interfere with the Min system of *B.subtilis* by introducing *yfp‐minD*_*Cd*_ fusion under the control of an IPTG‐inducible promoter (P_hyperspank_) into an *amyE* locus, creating strain IB1415. We assumed that YFP‐MinD_Cd_ could functionally substitute for the native MinD_Cd_, as both GFP‐MinD_Bs_ and GFP‐MinD_Ec_ are functional in their respective native organisms (Pavlendová et al. 2010; Raskin and de Boer 1999a). Cell length measurements were performed without IPTG and with two different IPTG concentrations, 0.1 mmol/L and 0.5 mmol/L. In the absence of inducer, the cell length of the strain harboring *yfp‐minD*_*Cd*_ (IB1415) was unchanged relative to the parental MO1099 strain (2.4 *μ*m; only 0.8% of cells are longer than 4 *μ*m) and no minicells were observed, suggesting that even though P_hyperspank_ is known to be leaky (Vavrová et al. 2010), the MinD_Cd_ amounts resulting from this leakiness are not sufficient to induce cell elongation. The addition of an inducer, regardless on the concentration, triggered elongation, with the average cell length reaching 4 *μ*m and 40% of cells becoming longer than this (Table S3). Additionally, we determined the cell length of a strain harboring MinE_Cd_, to verify the effect of MinE_Cd_ alone on cell division. To prepare a *B.subtilis* strain producing MinE_Cd_ (IB1410), we placed the corresponding gene under the control of a xylose‐inducible promoter (P_*xyl*_) into a *thrC* locus. We observed no notable change in average cell length (2.3–2.5 *μ*m) regardless of the presence of inducer at either concentration (Table S3). This is the same behavior we observed for MinE_Ec_ in a previous study (Pavlendová et al. 2010). In *E.coli,* MinE_Ec_ overexpression is characterized by the production of minicells (de Boer et al. 1989), but neither MinE_Cd_ nor MinE_Ec_ seemed to induce their formation when introduced into *B.subtilis* cells.

Finally, we assessed the effects of simultaneous MinD_Cd_E_Cd_ expression on the length of *B.subtilis* cells. A strain harboring both MinD_Cd_ and MinE_Cd_ (*yfp‐minD*_*Cd*_ *minE*_*Cd*_; IB1417) was prepared by transformation using chromosomal DNA as described in the Experimental procedures and Table 1. In the absence of inducers, IB1417 cells retained the same length as the parental wild‐type strain MO1099 (2.5 *μ*m; Table S3). Both induction conditions lead to comparable elongation, with average cell length that exceeded 3.7 *μ*m and 33% of cells were longer than 4 *μ*m (Table S3). Apparently, increasing the inducer concentration, and thus the amounts of MinD_Cd_ and MinE_Cd_, does not further increase cell length. Taken together, these results show that MinC_Cd_ and MinD_Cd_, but not MinE_Cd_, elongate cells and induce minicell formation when overexpressed in *B.subtilis*. Elongation was slightly less distinct when both MinD_Cd_ and MinE_Cd_ were coexpressed at low inducer concentrations.

### Complementation of the *B.subtilis* Min system with *C.difficile* Min proteins

It is known that the absence of MinC, MinD, or both in *B.subtilis* causes a slight cell elongation and the formation of minicells (Levin et al. 1992, 1998). There are several studies showing that the Min proteins of one organism can complement the function of the Min system of a different organism (Szeto et al. 2001; Tavva et al. 2006). For example, a functional exchange of Min systems between gram‐negative and gram‐positive bacteria showed that the expression of a heterologous *E.coli* MinD_Ec_ protein was able to partially rescue the *ΔminD*_*Bs*_ phenotype of *B.subtilis*; however, the same could not be said for *E.coli* MinC_Ec_, which failed to improve the *ΔminC*_*Bs*_ phenotype (Pavlendová et al. 2010).

Here, we investigated whether the MinC_Cd_ and MinD_Cd_ proteins of the *C.difficile* Min system could restore defects caused by deleting their homologues in *B.subtilis*, and whether coexpressing MinD_Cd_ and MinE_Cd_ together could restore defects caused by the absence of MinD_Bs_. If MinC_Cd_ or MinD_Cd_ do complement MinC_Bs_ and MinD_Bs_, the cells should become shorter and minicell formation should decrease. Previously utilized constructs with respective *C.difficile* genes under the control of inducible promoters were introduced into various *B.subtilis* mutant backgrounds. These strains and their complete genotypes are listed in Table 1. The average cell lengths of the resulting strains were measured in the presence of varying inducer concentrations and compared with those of their parental mutant strains. All of the following measurements are summarized in Table S3 and are illustrated in histogram in Figure 1. The length of parental strains *B.subtilis ΔminD*_*Bs*_ (IB1056) and *ΔminC*_*Bs*_ (IB1141) were both determined to be 4 *μ*m on average, with 45% of cells being longer than that.

To investigate the ability of MinC_Cd_ to complement the absence of MinC_Bs_, we created a strain producing MinC_Cd_ from a xylose‐inducible promoter (P_*xyl*_) in a *ΔminC*_*Bs*_ background (IB1550). The experiments were performed without xylose induction and with two different xylose concentrations (0.02% and 0.3%). Complementation effect was already observed in the absence of inducer, when leaky expression of MinC_Cd_ was sufficient to shorten the cells from 4.1 *μ*m to 3.4 *μ*m on average. Induced expression improved the phenotype even further, causing the percentage of cells longer than 4 *μ*m drop from 45% to 12% (Table S3). Minicells were present in all samples of *ΔminC*_*Bs*_ *minC*_*Cd*_.

Introducing a YFP‐MinD_Cd_ into a *ΔminD*_*Bs*_ background (IB1056) yielded strain *ΔminD*_*Bs*_ *yfp‐minD*_*Cd*_ (IB1416). The experiments were carried out without IPTG and with two different IPTG concentrations, 0.1 mmol/L IPTG and 0.5 mmol/L IPTG. Measurements of strain IB1416 grown without inducer produced cells with an average length similar to that of the originating *ΔminD*_*Bs*_ strain (Table S3). The low levels of MinD_Cd_ due to leaky expression therefore appeared to have no visible effect on cell length. Moderate expression of MinD_Cd_ (induction with 0.1 mmol/L IPTG) seemed to have a slight effect on cell division, as cell length decreased to 3.6 *μ*m and the proportion of cells longer than 4 *μ*m went down to 28%. Increasing the concentration of inducer to 0.5 mmol/L IPTG, however, lead to cell elongation (average of 4 *μ*m and 43% of cells longer than 4 *μ*m), just as seen during overexpression on a wild‐type background (IB1415) (Table S3). Minicells, which are a phenotype of both *ΔminD*_*Bs*_ mutation and also, as we have shown here, MinD_Cd_ overexpression, were observed in all samples, but their frequency was not evaluated.

We also investigated changes in cell length when MinD_Cd_ is expressed together with MinE_Cd_ in a strain lacking MinD_Bs_ (*ΔminD*_*Bs*_ *minD*_*Cd*_ *minE*_*Cd*_; IB1418). In this strain, only the induced expression of MinD_Cd_E_Cd_ decreased both cell length and the proportion of cells longer than 4–3.5 *μ*m and 29%, respectively, when using a lower induction level (0.1 mmol/L IPTG, 0.02% xylose), and to 3.3 *μ*m and 21% when using a higher induction level (0.5 mmol/L IPTG, 0.3% xylose) (Table S3).

In conclusion, our results suggest that MinC_Cd_ is able to complement for the absence of MinC_Bs_. MinD_Cd_ alone can only partially substitute for a missing MinD_Bs_, but when coexpressed with MinE_Cd_, considerably enhanced complementation is observed.

### Localization of *C.difficile* MinD and MinE in *B.subtilis*

**Figure 2:**
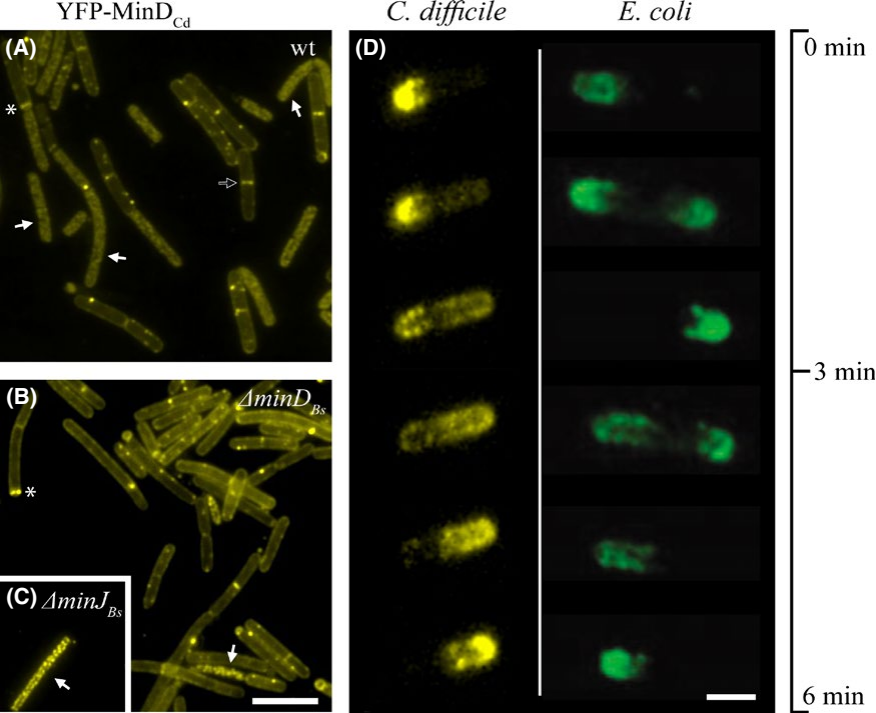
Localization and oscillation of *C.difficile* Min proteins. (A)–(C) Localization of YFP‐tagged MinD_C__d_ expressed from P_*hyperspank*_ in wild‐type and mutant *B. subtilis* backgrounds. (A) wt (IB1415), (B) *∆min* *D*_*B*__*s*_ (IB1416), (C) *∆min* *J*_*B*__*s*_ (IB1553). Full arrows point to examples of cells where the fine‐structure signal resembles the localization pattern of the native MinD_B__s_ along lipid spirals; the empty arrow indicates an example of localization to the vegetative septum, and the asterisk, to the asymmetric septum. Expression was induced with 0.1 mM IPTG; the scale bar represents 5 *μ*m. (D) Time‐lapse images recorded over a period of 6 min showing oscillation of MinD_C__d_MinE_C__d_ in a *B.subtilis ∆min* *D*_*B*__*s*_ background (IB1418), compared to oscillation of *E.coli* proteins in a *B.subtilis ∆min* *D*_*B*__*s*_ background (Jamroškovič et al. 2012). Expression of MinD_C__d_ was induced with 0.1 mM IPTG and MinE_C__d_ with 0.02% xylose; scale bar represents 1 *μ*m. Available also as Video S1 in Supporting Information.

As observation using fluorescent proteins is not yet commonly feasible in the anaerobic *C.difficile*, we explored the localization of its Min proteins in a heterologous *B.subtilis* system, which has previously proven to be a suitable environment for the study of Min proteins. We introduced a YFP‐MinD_Cd_ fusion into *B.subtilis* cells under the control of an IPTG‐inducible promoter at the *amyE* locus. This fusion was introduced into wild‐type, *ΔminD*_*Bs*_ and *ΔminJ*_*Bs*_ mutant backgrounds creating strains IB1415, IB1417, and IB1553, respectively. As expected from the similarity of MinD_Cd_ to MinD_Bs_, MinD_Cd_ localized to the cell membrane and often formed foci (Figs. 2A–C). The localization pattern between the wild‐type and mutant strains did not differ and we often observed short helical‐like structures resembling those seen previously with *B.subtilis* MinD (Barák et al. 2008). In many instances, we observed localization to the sites of vegetative and asymmetric septa as well as the polar sites (Figs. 2A and B).

The localization of MinE_Cd_ was examined using a MinE_Cd_‐GFP fusion placed under the control of a xylose‐inducible promoter in a wild‐type background (IB1552). The observed signal was dispersed throughout the cytoplasm (not shown), which is similar to the localization of *E.coli* MinE_Ec_‐GFP in *B.subtilis* observed previously (Pavlendová et al. 2010).

### Oscillation of *C.difficile* MinDE proteins in *B.subtilis*

We have previously shown that the oscillation of the *E.coli* Min system can be reproduced in *B.subtilis* (Jamroškovič et al. 2012). Because manipulation of an anaerobic pathogenic bacteria poses a number of complications, we decided to explore the behavior of the *C.difficile* Min proteins in *B.subtilis* cells. We introduced MinE_Cd_, under the control of a xylose‐inducible promoter at the *thrC* locus, into strains already harboring a YFP‐MinD_Cd_ fusion at the *amyE* locus. This gave rise to strains carrying the *C.difficile* genes *MinD*_*Cd*_ and *MinE*_*Cd*_ in wild‐type, *ΔminD*_*Bs*_ and *ΔminD*_*Bs*_ *ΔminJ*_*Bs*_ *,* mutant backgrounds (strains IB1417, IB1418 and IB1546). When these proteins were coexpressed using 0.1 mmol/L IPTG and 0.02% xylose on the wild‐type background (IB1417), we observed oscillation of YFP‐MinD_Cd_ from one pole to the other in a small number of cells (roughly 3% of 250 cells). This is in contrast with the behavior of the corresponding *E.coli* proteins in *B.subtilis*, which showed no oscillation at all in the wild‐type. This effect was probably due to an interaction between MinD_Bs_ and MinD_Ec_ (Jamroškovič et al. 2012).

In *E.coli*, Min oscillation cycle period is 20–50 sec (Raskin and de Boer 1999a; Touhami et al. 2006); the oscillation of the *E.coli* proteins in *B.subtilis* is somewhat slower, with a period of 1.5–3 min (recorded against a *∆minD*_*Bs*_ *∆divIVA*_*Bs*_ background; IB1242), and increasing the temperature to 30°C or 37°C does not affect the oscillation speed (Jamroškovič et al. 2012). Strain *ΔminD*_*Bs*_ *ΔdivIVA*_*Bs*_ *yfp‐minD*_*Ec*_ *minE*_*Ec*_ (IB1242) was used as a control strain in this study, to ensure our conditions are properly set, as it showed the most extensive oscillation of *E.coli* Min proteins in *B.subtilis*.

The oscillation of the *C.difficile* proteins observed against a wild‐type *B.subtilis* background (IB1417) was even slower than the *E.coli* ones, at a pace of about 3–5 min per cycle at room temperature. The oscillation period in strains depleted of MinD_Bs_ or both MinD_Bs_ and MinJ_Bs_ did not change and remained at 3–5 min per cycle. However, the absence of these components seemed to increase the proportion of cells in which oscillation was observed. In a strain lacking MinD_Bs_ (IB1418, Fig. 2D, Video S1), the effect was similar to the wild‐type strain (4% of 50 cells), but when both MinD_Bs_ and MinJ_Bs_ were absent (IB1546), the oscillation was observed in up to 50% of the cells (Video S2). Regardless of the strain observed, the oscillation often stopped after 10 min and the YFP signal became dispersed throughout the cell. The speed and extent of oscillation for various organisms and heterologous systems is summarized in Table 2.

**Table 2:** Comparison of oscillation times and efficiency between Min systems

System	Organism	Genotype	Oscillation efficiency [%]	Oscillation period	Reference
*E.coli*	*E.coli*	–	100	20–50 sec	Raskin and de Boer 1999a; Touhami et al. 2006
*E.coli*	*B.subtilis*	*YFP‐minD*_*Ec*_ *minE*_*Ec*_	0	–	Jamroškovič et al. 2012
*E.coli*	*B.subtilis*	*∆minD*_*Bs*_ *∆divIVA*_*Bs*_ *YFP‐minD*_*Ec*_ *minE*_*Ec*_	~100	1.5–3 min	Jamroškovič et al. 2012
*C.difficile*	*B.subtilis*	*YFP‐minD*_*Cd*_ *minE*_*Cd*_	3	3–5 min	This study
*C.difficile*	*B.subtilis*	∆*minD*_*Bs*_ *YFP‐minD*_*Cd*_ *minE*_*Cd*_	4	3–5 min	This study
*C.difficile*	*B.subtilis*	∆*minD*_*Bs*_ *∆minJ*_*Bs*_ *YFP‐minD*_*Cd*_ *minE*_*Cd*_	50	3–5 min	This study

To determine if *C.difficile* MinE could drive oscillation of MinD of *B.subtilis*, we prepared a strain carrying a combination of GFP‐tagged MinD_Bs_ and MinE_Cd_ in the *B.subtilis ΔminD*_*Bs*_ *ΔminJ*_*Bs*_ background which showed the most efficient oscillation of MinD_Cd_E_Cd_. Fluorescence microscopy of this strain (*ΔminD*_*Bs*_ *ΔminJ*_*Bs*_ *gfp‐minD*_*Bs*_ *minE*_*Cd*_; IB1562) revealed that no oscillation or movement of foci could be observed after induction with 0.04% xylose (not shown). The GFP signal was distributed as random foci throughout the cell and along the helical structures which are characteristic of MinD_Bs_ (Barák et al. 2008).

### Swap of Min system oscillating components between *C.difficile* and *E.coli*

To investigate the interchangeability of the Min proteins from the gram‐positive *C.difficile* and the gram‐negative *E.coli* and their ability to oscillate together, we prepared *B.subtilis* strains *ΔminD*_*Bs*_ *yfp‐minD*_*Ec*_ *minE*_*Cd*_ (IB1412) and *ΔminD*_*Bs*_ *ΔdivIVA*_*Bs*_ *yfp‐minD*_*Ec*_ *minE*_*Cd*_ (IB1413). After inducing the expression of YFP‐MinD_Ec_ and MinE_Cd_, we observed oscillation of YFP‐MinD_Ec_ with a period of 3–5 min, similar to that observed before for strain expressing MinDE originating from *C.difficile* (IB1417). This oscillation was only observed in a small portion of the *ΔminD*_*Bs*_ cells (IB1412) and improved in cells with a *ΔminD*_*Bs*_ *ΔdivIVA*_*Bs*_ background (IB1413; not quantified statistically). These results show that there is some compatibility between the oscillating systems of these two evolutionarily distant gram‐positive and gram‐negative species.

### Oscillating Min system of *C.difficile* interferes with *B.subtilis* sporulation

**Figure 3:**
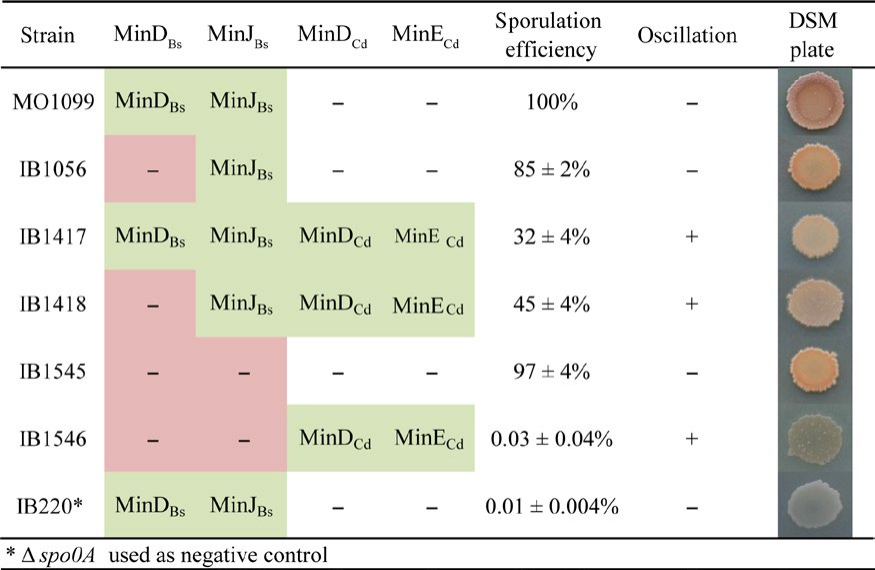
Sporulation efficiency of *B.subtilis* strains. Sporulation efficiency is given as the mean ± SD of at least three independent assays, each normalized against a wild‐type control. Sporulating colonies develop brown color, while nonsporulating light are brown to translucent, as seen in the *∆spo0A* negative control (IB220).

In our previous study, we showed that the oscillating *E. coli* Min system blocks sporulation at the asymmetric septum formation step (Jamroškovič et al. 2012). An intriguing question is therefore whether spore‐forming *C.difficile* also possesses an oscillating Min system that interferes with its sporulation. We assessed the sporulation efficiency of various *B.subtilis* strains in the presence of inducers (0.5 mmol/L IPTG, 0.5% xylose). The sporulation efficiency of both the wild‐type and a *ΔminD*_*Bs*_ strain harboring the oscillating *C.difficile* MinDE proteins (IB1417 and IB1418) dropped to 32% and 45%, respectively (Fig. 3). A dramatic decrease in sporulation efficiency, down to 0.03%, was observed in the strain which lacked both MinD_Bs_ and MinJ_Bs_ (IB1546). This was also the strain with the most effective oscillation. This drop in sporulation efficiency seems to be related to the proportion of cells in which oscillation is observed.

### Protein–protein interactions between *C.difficile* MinD and *B.subtilis* Min proteins

**Figure 4:**
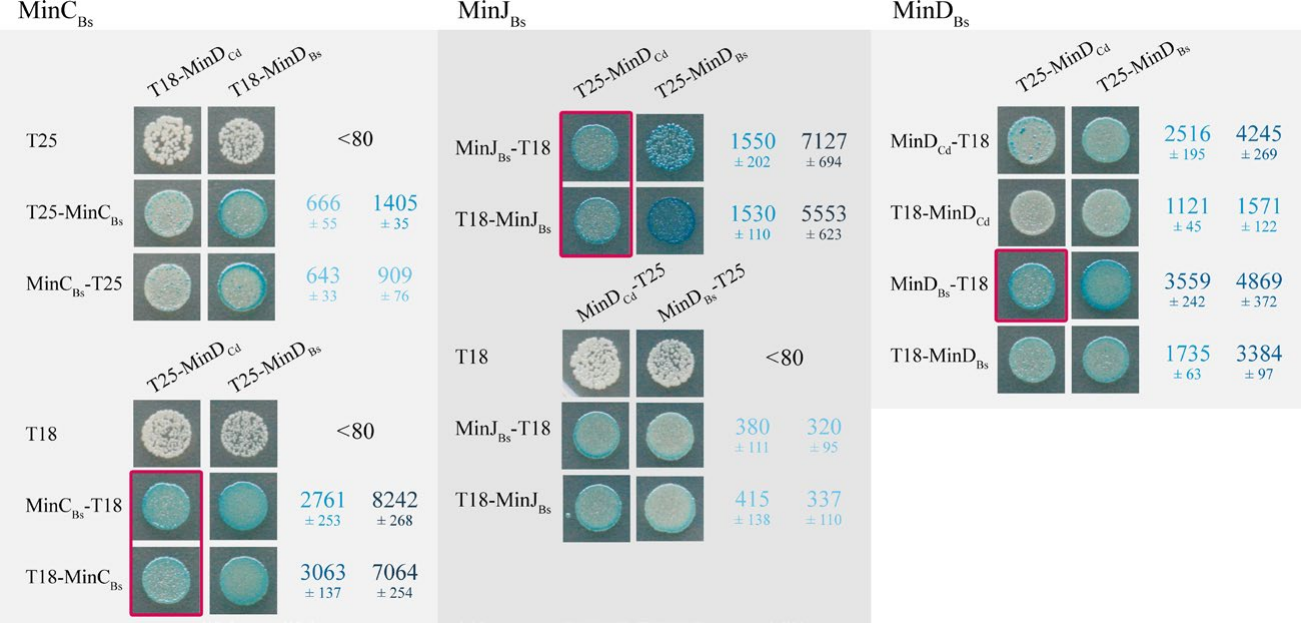
Protein–protein interactions between MinD_C__d_ and the *B.subtilis* Min proteins compared alongside interactions among the *B.subtilis* Min proteins as detected by bacterial two‐hybrid system BACTH. Interactions were quantified using a *β*‐galactosidase assay and are expressed in Miller units as mean values ± SD of at least three independent measurements. The color intensity corresponds to the strength of the interaction; red boxes highlight strong positive interactions between heterologous proteins. Negative controls were all below 80 MU.

To improve our understanding of the behavior of the *C.difficile* Min proteins in *B.subtilis,* we looked for interactions between the *C.difficile* and *B.subtilis* proteins using a bacterial two‐hybrid system (BACTH). The strength of these interactions was quantified using a *β‐*galactosidase assay. A very strong interaction was detected between MinD_Cd_ and MinC_Bs_, while a weaker one was found between MinD_Cd_ and MinJ_Bs_ (Fig. 4). It is possible that the lower affinity of MinD_Cd_ for MinJ_Bs_ might explain why only partial complementation was observed when MinD_Cd_ was expressed against a *ΔminD*_*Bs*_ background (IB1416), as the MinD–MinJ interaction would clearly be a limiting factor. The MinD proteins from the two organisms seem to interact with each other strongly as well. The interaction observed between MinD_Cd_ and MinC_Bs_ confirms that the MinD_Cd_/MinE_Cd_ system can indeed utilize the host *B.subtilis* MinC, as suggested by the complementation experiments in the *ΔminD*_*Bs*_ *minD*_*Cd*_ *minE*_*Cd*_ (IB1418) strain.

## Discussion

*Clostridium difficile* is an important human pathogen, causing serious problems in hospitals and medical facilities (reviewed in Burke and Lamont 2014). Because of its strictly anaerobic life style and its demanding transformation procedures, we have still only a limited knowledge of some of its basic processes, including cell division. For example, commonly used reporter genes, such as fluorescent proteins or luciferase, require oxygen for protein folding or enzyme activity (Heim et al. 1994; Hastings and Gibson 1967). In spite of ongoing efforts, methods and reporter assays suitable for anaerobic or low‐oxygen conditions are only now starting to emerge (Drepper et al. 2007; Edwards et al. 2015; Buckley et al. 2015; Ransom et al. 2015). Because of these problems, we decided to investigate the mechanism of action of *C.difficile* Min proteins and their effects on vegetative cell division and sporulation in a heterologous *B.subtilis* host. This, the first study focused on the *C.difficile* Min system, may help us to understand the role of its Min proteins in the asymmetric division and spore formation of this medically significant bacterium.

**Figure 5:**
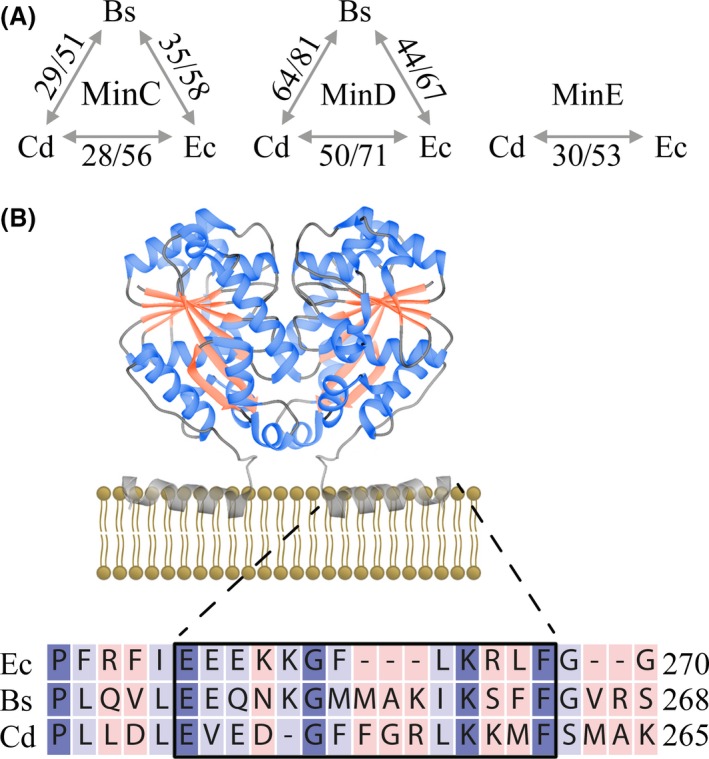
Bioinformatic analysis of Min_Cd_ proteins. (A) Percent sequence identity (same residues)/similarity (same residues + positive substitutions) between the Min proteins of *B.subtilis* (Bs), *C.difficile* (Cd) and *E.coli* (Ec) based on data from BLAST search (Altschul et al. 1997). (B) Model of the attachment of a MinD_E__c_ dimer to the membrane interface through its amphipathic helix. This model is based on the MinD_E__c_ crystal structure (PDB ID: 3Q9L), which contains residues 1–260, and thus lacks some of the residues involved in helix formation. Below: Alignment of the C‐terminal region of MinD containing the amphipathic helix. The consensus region is boxed, identical residues are violet, and conservation is indicated by color intensity.

Our analysis of MinC_Cd_ and MinD_Cd_ in vegetatively growing cells shows that these proteins are able to affect cell division in *B.subtilis*. We found that both MinC_Cd_ and MinD_Cd_ can complement for the missing *B.subtilis* counterparts. Interestingly, the same could not be said for MinC_Ec_, which was previously shown to fail in MinC_Bs_ complementation (Pavlendová et al. 2010), and which has higher similarity to MinC_Bs_ (35/58% identity/similarity based on BLAST alignment) than MinC_Cd_ has (29/51% identity/similarity; Fig. 5A, Fig. S1). As MinD_Bs_ is more similar to MinD_Cd_ than it is to MinD_Ec_ (64/81% identity/similarity compared to 44/67%; Fig. 5A, Fig. S1), we might expect MinD_Cd_ to better complement a MinD_Bs_ deletion than MinD_Ec_ (Pavlendová et al. 2010), however, this is not what we observed. The *B.subtilis* Min system is finely tuned, with relatively small changes having clearly notable effects, so it is not surprising that substituting one of the components with a replacement that has different binding affinities for all the other elements involved with the system leads to divergent effects, a feature which might not be reflected or predicted solely by the sequence similarity. More importantly, the complementation of MinD_Bs_ absence by MinD_Cd_/MinE_Cd_ coexpression revealed that this oscillating Min system can still aid in proper septum placement when the native Min system is disturbed, provided that these proteins can engage the native system's MinC.

The observed localization of MinD_Cd_ along helical structures suggests that this protein recognizes the anionic phospholipids organized in a helical manner in the *B.subtilis* membrane. The C‐terminal region of MinD from various organisms, including *E.coli* and *B.subtilis*, contains a consensus amphipathic helical region that anchors it to the membrane (Szeto et al. 2002). This consensus sequence can also be found in *C.difficile* MinD (Fig. 5B) and an amphipathic helix was also predicted at the C‐terminus by AmphipaSeek (not shown; Sapay et al. 2006). This helix would then be responsible for the membrane localization of MinD_Cd_ in *B.subtilis,* which is similar to that observed for MinD_Ec_ in the same organism.

*Bacillus subtilis* and *C.difficile* have very different membrane compositions, In fact, *Clostridium* species display distinct variations in their major polar lipid contents and *C.difficile* has an exceptionally variable membrane lipid composition, even for different isolates of the same strain (Korachi et al. 2002). Phosphatidylglycerol (PG) and cardiolipin (CL) have been identified, but interestingly, phosphatidylethanolamine is completely absent and other lipids have been proposed to balance the negative charge of PG and CL (Drucker et al. 1996; Korachi et al. 2002; Guan et al. 2014). Phosphatidylglycerol and CL represent 24% and 4% of the membrane phospholipids in *E.coli* (Kusters et al. 1991), 40% and 20% in *B.subtilis* (López et al. 1998), and 30% and 16% in *C.difficile* (Guan et al. 2014).

The ability to oscillate is an intrinsic characteristic of the Min proteins and emerges whenever some minimal criteria are met (Loose et al. 2008). Thus, a different membrane composition does not pose any obstacle for oscillation, as long as enough negatively charged lipids are present, although the resulting charge density does affect oscillation parameters such as wavelength and velocity (Vecchiarelli et al. 2014; Zieske and Schwille 2014). It has been suggested that these differences arise from the differences between the mechanisms of membrane binding by MinD_Ec_ and MinE_Ec_ (Vecchiarelli et al. 2014). Previous successful reconstitutions of oscillation in heterologous systems (Ramirez‐Arcos et al. 2002; Jamroškovič et al. 2012) suggest that, in the complex environment of cell, the most limiting factor is the interaction between the heterologous Min proteins within the host organism. In our case, oscillation markedly improved when the Min_Cd_ system was introduced into a *ΔminD*_*Bs*_ *ΔminJ*_*Bs*_ background (IB1546), which allowed oscillation of the heterologous Min system.

All *B.subtilis* strains expressing an oscillating Min_Cd_ system exhibited disturbed sporulation. The severity of the sporulation defect seems to be correlated with improved oscillation efficiency. Two important questions remain: first, what is the underlying cause of this failed sporulation, and second, does it affect the sporulation of *C.difficile* at all? It is possible that the differences in sporulation and sporulation regulation between *B. subtilis* and *C.difficile* cause oscillation to be inhibitory in the heterologous organism, but not in the native one. Another possibility is that an oscillating Min_Cd_ system only inhibits polar division during particular parts of the cell life cycle, such as vegetative growth, and is shut down or modulated during sporulation. Yet, a third possibility is that this system does, in fact, cause *C.difficile* to sporulate less efficiently than *B.subtilis*, but provides additional advantage for its different lifestyle and environmental niche.

Although complex transcriptional data for *C.difficile* are still lacking, we can still make some inferences from the work of Saujet et al. (2013). They found that the *ftsZ*,* minC*,* minD*,* minE*, and *divIVA* genes were all positively controlled by SigH, the key regulator of the transition phase in *C.difficile*, which is of comparable importance to Spo0A. SigH is also involved in the expression of *ftsZ*,* minC*, and *minD* in *B. subtilis* as well (Britton et al. 2002). These results suggest that the MinCDE proteins are present in the early stages of sporulation in *C.difficile*. The genome of *C.difficile* also harbors a DivIVA homolog (Table. S4), which in *B.subtilis* has a role in sporulation, but the question of whether it serves as a polar tether for the Min system as it does in *B.subtilis* remains open, as we were not able to identify a MinJ homologue. It is still possible that some other protein fills the role of MinJ in connecting the MinCD system to DivIVA.

The Clostridia are a diverse group of bacteria, and, despite their common historical designation as gram‐positive, a number of them have been found to have a membrane organization more characteristic of gram‐negative bacteria (and were thus moved into a separate class, *Negativicutes*), together with the ability to form endospores (Yutin and Galperin 2013). *Acetonema longum* is a distant relative of *Clostridium* spp. and a lesser known member of *Negativicutes*. A study of the sporulation and germination of this organism revealed a remarkable inversion of the inner membrane of the mother cell, to become the outer membrane of the germinating cell (Tocheva et al. 2011). This brings us to the question of evolution of gram‐positive and gram‐negative bacteria, an exciting topic on which many opposing theories exist. The work of Tocheva et al. 2011suggests how the outer membrane of gram‐negative bacteria might have evolved, and more broadly, how gram‐negatives could have arisen from gram‐positives. *A.longum* could therefore represent a missing link between the two groups. Our analysis of some Clostridia and Negativicutes members' genomes shows that many possess Min proteins from both systems (Table. S4), suggesting that the two systems might have evolved in a gram‐positive bacterium. Whether and how these systems could coexist in clostridia remains to be resolved by future studies. Until convenient methods for directly studying clostridia are developed, *B.subtilis* could serve as host system for these studies.

## Conflict of Interest

Authors declare no conflict of interest.

## Supporting information


**Figure S1:** Multiple sequence alignment of Min proteins.

**Video S1:** Oscillation of YFP‐tagged MinD_Cd_ in the presence of MinE_Cd_, recorded in *B.subtilis ΔminD*_*Bs*_ *minD*_*Cd*_ *minE*_*Cd*_ (IB1418). Scale bar represents 1 μm.

**Video S2:** Oscillation of YFP‐tagged MinD_Cd_ in the presence of MinE_Cd_, recorded in *B.subtilis ΔminD*_*Bs*_ *ΔminJ*_*Bs*_ *minD*_*Cd*_ *minE*_*Cd*_ (IB1546). Scale bar represents 5 *μ*m.

**Table S1:** Plasmids used in this study and their construction.
**Table S2:** Primers used in this study.
**Table S3:** Cell length measurements.
**Table S4:** The sequence similarity/identity of Min proteins of selected members of Clostridia and Negativicutes compared with their counterparts in *E.coli* and *B.subtilis*. Similarity and identity values are derived from a BLAST query (Altschul et al. 1997). For MinC and MinD, the sequence of the *B.subtilis* proteins was used as reference, since these queries gave lower E‐values and higher query cover and identities than the *E.coli* sequences (not shown). The *E.coli* sequence was used as a reference for MinE, and *B.subtilis* sequences for a search of MinJ and DivIVA homologs. All listed organisms are endospore‐formers except *E.coli*.
