# Supplementary figures and images for: Oscillating behavior of Clostridium difficile Min proteins in Bacillus subtilis
Source: Microbiologyopen. 2016 Jan 27;5(3):387–401. doi: 10.1002/mbo3.337 (PMC4905992; doi:10.1002/mbo3.337)

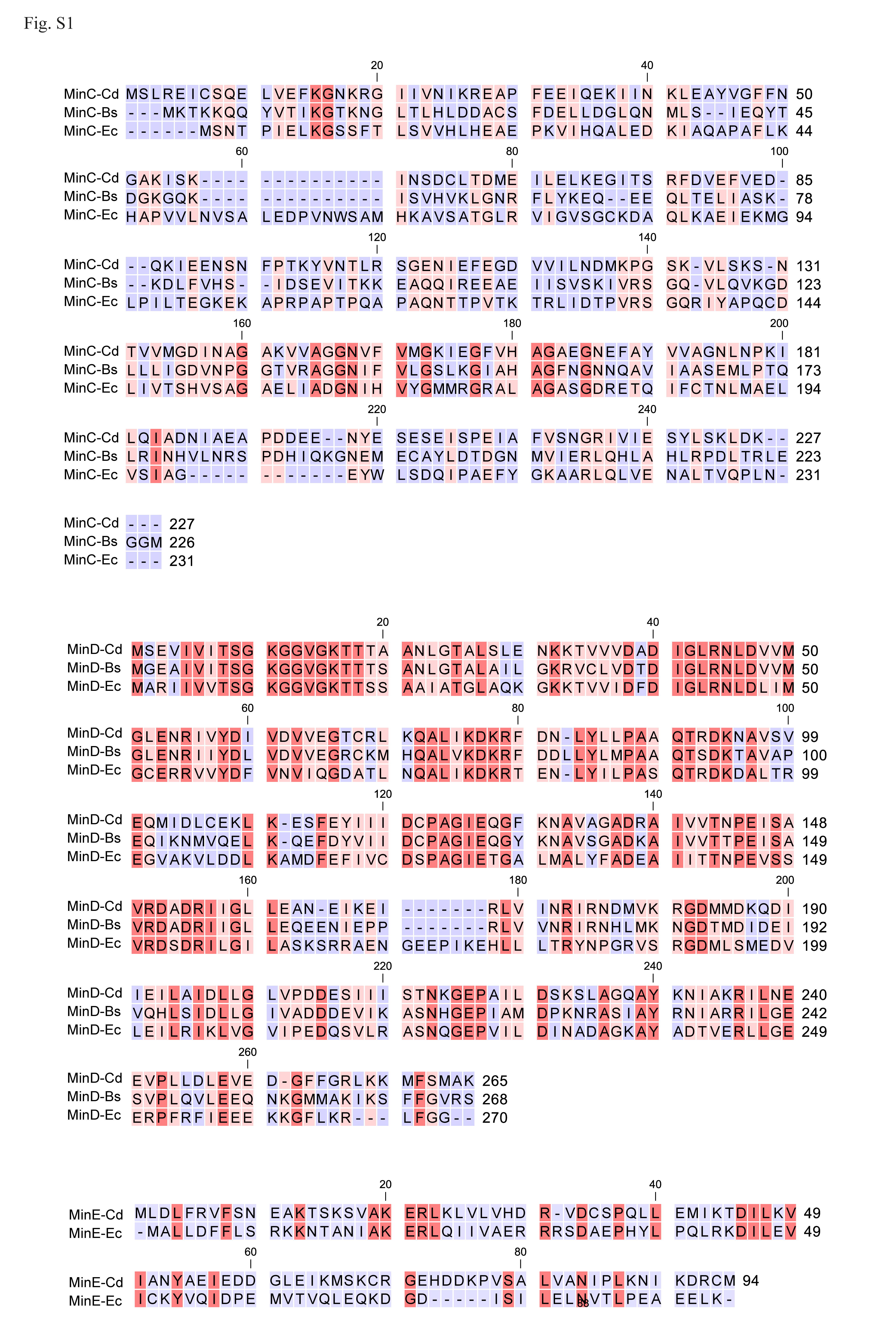

Supplement: Supplementary file 1 — Figure S1: Multiple sequence alignment of Min proteins. [file MBO3-5-387-s001.tif]
