# Supplementary material for: Oscillating behavior of Clostridium difficile Min proteins in Bacillus subtilis
Source: Microbiologyopen. 2016 Jan 27;5(3):387–401. doi: 10.1002/mbo3.337 (PMC4905992; doi:10.1002/mbo3.337)
Supplement: Supplementary file 4 — Table S1: Plasmids used in this study and their construction. Table S2: Primers used in this study. Table S3: Cell length measurements. Table S4: The sequence similarity/identity of Min proteins of selected members of Clostridia and Negativicutes compared with their counterparts in E.coli and B.subtilis. Similarity and identity values are derived from a BLAST query (Altschul et al. 1997). For MinC and MinD, the sequence of the B.subtilis proteins was used as reference, since these queries gave lower E‐values and higher query cover and identities than the E.coli sequences (not shown). The E.coli sequence was used as a reference for MinE, and B.subtilis sequences for a search of MinJ and DivIVA homologs. All listed organisms are endospore‐formers except E.coli. [file MBO3-5-387-s004.docx]

**Supporting Information**

**Table S1:** Plasmids used in this study and their construction.

| **Plasmids** | **Description/Construction** | **Reference** |
| --- | --- | --- |
| pED962 | Vector for IPTG-inducible expression of gene of interest in *B. subtilis*. | D. Rudner  (unpublished data) |
| pED-yfp-minD_Cd_ | Plasmid for IPTG-inducible expression of YFP-MinD_Cd_ in *B. subtilis*. *minD_Cd_* was amplified by PCR using oligos MinD_Cd_SalIS/MinD_Cd_SphIE. The PCR fragment was cloned into pED962 using SalI/SphI restriction sites. | This study |
| pNP1 | Vector for xylose-inducible expression of gene of interest in *B. subtilis*. | Jamroškovič *et al.* (2012) |
| pNP-minE_Cd_ | Plasmid for xylose-inducible expression of MinE_Cd_ in *B. subtilis*.  *minE_Cd_* was amplified by PCR using oligos MinE_Cd_KpnIS/MinE_Cd_XhoIE. The PCR fragment was cloned into pNP1 using KpnI/XhoI restriction sites | This study |
| pSGmGFP | Vector harbouring a xylose-inducible promoter followed by a gene encoding monomeric GFP (mGFP). | gift from Henrik Stahl |
| pSGminE_Cd_mGFP | Plasmid for xylose-inducible expression of MinE_Cd_-mGFP in *B. subtilis*.  *minE_Cd_* was amplified by PCR using oligos MinEcdKpnIS/MinEcdKpnIE and cloned into pSGmGFP using a KpnI restriction site. | This study |
| pSG1729 | Vector harbouring a xylose-inducible promoter followed by a gene encoding GFP. | Lewis & Marston (1999) |
| pSGminC_Cd_ | Plasmid for xylose-inducible expression of MinC_Cd_ in *B. subtilis*.  *minC_Cd_* was amplified by PCR using oligos MinC_Cd_dKpnIS/MinC_Cd_EcoRIE and cloned into pSG1729 outside *gfp* reading frame using KpnI/EcoRI restriction sites. | This study |
| pUT18 | Vector for N-terminal fusion of T18 domain of adenylate-cyclase with desired protein, for use in bacterial two-hybrid analysis (BACTH). | Karimova *et al.*, 1998 |
| pUT18C | Vector for C-terminal fusion of T18 domain of adenylate-cyclase with desired protein, for use in BACTH. | Karimova *et al.*, 1998 |
| pKNT25 | Vector for N-terminal fusion of T25 domain of adenylate-cyclase with desired protein, for use in BACTH. | Karimova *et al.*, 1998 |
| pKT25 | Vector for C-terminal fusion of T25 domain of adenylate-cyclase with desired protein, for use in BACTH. | Karimova *et al.*, 1998 |
| pUT-minD_Cd_ | *minD_Cd_* was amplified by PCR using primer pair minDcdB2HF-BamHI-START/minDcdB2HR-EcoRI-NOSTOP and cloned into pUT18 using EcoRI/BamHI sites | This study |
| pUTC-minD_Cd_ | *minD_Cd_* was amplified by PCR using primer pair minDcdB2HF-BamHI-NOSTART/minDcdB2HR-EcoRI-STOP and cloned into pUT18C using EcoRI/BamHI sites | This study |
| pKT-minD_Cd_ | *minD_Cd_* was amplified by PCR using primer pair minDcdB2HF-BamHI-NOSTART/minDcdB2HR-EcoRI-STOP and cloned into pKT25 using EcoRI/BamHI sites | This study |
| pKNT-minD_Cd_ | *minD_Cd_* was amplified by PCR using primer pair minDcdB2HF-BamHI-START/minDcdB2HR-EcoRI-NOSTOP and cloned into pKNT25 using EcoRI/BamHI sites | This study |

**Table S2:** Primers used in this study.

| **primer** | **Sequence (5′–3′), restriction sites are underlined** |
| --- | --- |
| minDcdSalIS | GATGATGATGATGTCGACGGAGTATCTATTGTAATAAC |
| minDcdSphIE | GATGATGATGATGCATGCTTAATTTCGCTTAAATAGCTTC |
| minEcdKpnIS | GATGATGATGATGGTACCGAGAGAATGGGTTTTTTTAAGAG |
| minEcdXhoIE | GATGATGATGATCTCGAGGTTACTTATCTTCCCTTTATAC |
| minEcdKpnIS | GACGAGGACGGTACCATGTTAGATTTATTTAGAG |
| minEcdKpnIE | CCTGTTAAGCACGGTACCCATACATCTATC |
| minCcdKpnIS | CACTCTGGAGGGTACCATGTCTTTAAGAG |
| minCcdEcoRIE | GCACCTAGAATTCGTTTTATTTGTCTAACTTTGAC |
| minDcdB2HF-BamHI-START | CTAGAATGGGATCCTATGAGCGAAGTTATAG |
| minDcdB2HR-EcoRI-NOSTOP | GTATCCTTGAATTCGACTTAGCCATGC |
| minDcdB2HF-BamHI-NOSTART | GATGATAGGGATCCCAGCGAAGTTATAG |
| minDcdB2HR-EcoRI-STOP | GATGTATCCTGAATTCTTACTTAGCCATGC |

**Table S3:** Cell length measurements.

| Strain | MinC_Bs_ | MinD_Bs_ | MinC_Cd_ | MinD_Cd_ | MinE_Cd_ | Xylose [% w/v] and/or IPTG [mM] | Average cell length±SD [μm] | Cells longer than 4 μm [%] |
| --- | --- | --- | --- | --- | --- | --- | --- | --- |
| heterologous expression of Min proteins | | | | | | | | |
| MO1099 | C_Bs_ | D_Bs_ | *-* | *-* | *-* | No | 2.4±0.5 | 0.2 |
| IB1549 | C_Bs_ | D_Bs_ | C_Cd_ | - | - | No | 3.0±0.8 | 11.6 |
| IB1549 | C_Bs_ | D_Bs_ | C_Cd_ | - | - | 0.30% | 2.9±0.8 | 10.2 |
| IB1415 | C_Bs_ | D_Bs_ | - | D_Cd_ | - | No | 2.4±0.5 | 0.8 |
| IB1415 | C_Bs_ | D_Bs_ | - | D_Cd_ | - | 0.1 mM | 4.0±1.4 | 40.2 |
| IB1415 | C_Bs_ | D_Bs_ | - | D_Cd_ | - | 0.5 mM | 3.9±1.4 | 37.7 |
| IB1410 | C_Bs_ | D_Bs_ | - | - | E_Cd_ | No | 2.3±0.5 | 0.2 |
| IB1410 | C_Bs_ | D_Bs_ | - | - | E_Cd_ | 0.02% | 2.5±0.6 | 1.3 |
| IB1410 | C_Bs_ | D_Bs_ | - | - | E_Cd_ | 0.30% | 2.3±0.5 | 0.1 |
| IB1417 | C_Bs_ | D_Bs_ | - | D_Cd_ | E_Cd_ | No | 2.5±0.6 | 1.0 |
| IB1417 | C_Bs_ | D_Bs_ | - | D_Cd_ | E_Cd_ | 0.1 mM; 0.02% | 3.7±1.1 | 33.9 |
| IB1417 | C_Bs_ | D_Bs_ | - | D_Cd_ | E_Cd_ | 0.5 mM; 0.3% | 3.8±1.1 | 38.9 |
| complementation | | | | | | | | |
| IB1141 | - | D_Bs_ | - | - | - | No | 4.1±1.3 | 45.3 |
| IB1550 | - | D_Bs_ | C_Cd_ | - | - | No | 3.4±0.9 | 23.3 |
| IB1550 | - | D_Bs_ | C_Cd_ | - | - | 0.02% | 3.1±0.9 | 12.1 |
| IB1550 | - | D_Bs_ | C_Cd_ | - | - | 0.30% | 3.2±0.8 | 12.3 |
| IB1056 | C_Bs_ | - | - | - | - | No | 4.0±1.2 | 45.5 |
| IB1416 | C_Bs_ | - | - | D_Cd_ | - | No | 3.9±1.0 | 39.7 |
| IB1416 | C_Bs_ | - | - | D_Cd_ | - | 0.1 mM | 3.6±0.9 | 28.2 |
| IB1416 | C_Bs_ | - | - | D_Cd_ | - | 0.5 mM | 4.0±1.5 | 42.7 |
| IB1418 | C_Bs_ | - | - | D_Cd_ | E_Cd_ | No | 3.9±1.6 | 39.6 |
| IB1418 | C_Bs_ | - | - | D_Cd_ | E_Cd_ | 0.1 mM; 0.02% | 3.5±1.1 | 28.8 |
| IB1418 | C_Bs_ | - | - | D_Cd_ | E_Cd_ | 0.5 mM; 0.3% | 3.3±1.0 | 21.4 |

**Table S4:** The sequence similarity/identity of Min proteins of selected members of Clostridia and Negativicutes compared with their counterparts in *E. coli* and *B. subtilis*. Similarity and identity values are derived from a BLAST query (Altschul *et al.*, 1997). For MinC and MinD, the sequence of the *B. subtilis* proteins was used as reference, since these queries gave lower E-values and higher query cover and identities than the *E. coli* sequences (not shown). The *E. coli* sequence was used as a reference for MinE, and *B. subtilis* sequences for a search of MinJ and DivIVA homologues. All listed organisms are endospore-formers except *E. coli*. The spore formation of *Syntrophomonas wolfei* was not confirmed, although it contains full set of genes for spore formation (Sieber *et al.*, 2010).

| **Organism** | **Taxid** | **MinC_Bs_** | **MinD_Bs_** | **MinE_Ec_** | **MinJ_Bs_** | **DivIVA_Bs_** |
| --- | --- | --- | --- | --- | --- | --- |
| *Bacillus subtilis PY79* | 1415167 | 100/100 | 100/100 | - | 100/100 | 100/100 |
| *Escherichia coli* str. *K-12* substr. *MG1655* | 511145 | 36/56 | 44/67 | 100/100 | - | - |
| *Clostridium acetobutylicum* | 272562 | 32/55 | 57/77 | 34/57 | 23/42 | 34/64 |
| *Clostridium beijerinckii NCIMB 8052* | 290402 | 33/57 | 55/77 | 36/60 | 22/40 | 34/62 |
| *Clostridium botulinum* | 413999 | 35/55 | 53/75 | 40/67 | - | 38/60 |
| *Clostridium kluyveri* | 431943 | 33/55 | 54/77 | 33/65 | 23/45 | 34/63 |
| *Clostridium ljungdahlii* | 748727 | 34/54 | 54/77 | 32/63 | 26/47 | 35/64 |
| *Clostridium novyi* | 386415 | 33/55 | 56/77 | 41/62 | 24/40 | 41/69 |
| *Clostridium perfringens* | 195103 | 35/53 | 57/77 | 32/59 | 25/41 | 35/62 |
| *Clostridium tetani* | 1231072 | 32/54 | 54/77 | 37/64 | 23/41 | 31/64 |
| *Clostridium difficile* (*Peptoclostridium difficile*) | 272563 | 29/51 | 64/81 | 30/53 | - | 40/65 |
| *Clostridium thermocellum* (*Ruminiclostridium thermocellum*) | 203119 | 36/56 | 64/82 | 28/58 | - | 42/68 |
| *Symbiobacterium thermophilum* | 292459 | 34/52 | 55/78 | 29/65 | - | 32/64 |
| *Syntrophomonas wolfei* | 335541 | 34/53 | 62/83 | 28/66 | 23/43 | 33/61 |
| *Thermoanaerobacter wiegelii* | 697303 | 37/57 | 64/83 | 34/57 | 24/42 | 43/76 |
| *Alkaliphilus oremlandii* | 350688 | 35/56 | 62/78 | 29/54 | - | 44/66 |
| *Desulfotomaculum reducens* | 349161 | 29/49 | 80/62 | 30/61 | 23/42 | 30/63 |
| *Acetonema longum* | 1009370 | - | 59/81 | 24/61 | 24/38 | 46/76 |

**Figure S1: Multiple sequence alignment of Min proteins.** Sequences were aligned using ClustalW plugin of CLC Sequence Viewer 7.6 software. Background color corresponds to conservation level – dark red indicates conserved residue, light red partially conserved, blue non-conserved residue.

**Movie S1: Oscillation of YFP-tagged MinD_Cd_ in the presence of MinE_Cd_**, recorded in *B. subtilis* *ΔminD_Bs_ minD_Cd_ minE_Cd_* (IB1418). Scale bar represents 1 μm.

**Movie S2:** **Oscillation of YFP-tagged MinD_Cd_ in the presence of MinE_Cd_**, recorded in *B. subtilis* *ΔminD_Bs_ ΔminJ_Bs_ minD_Cd_ minE_Cd_* (IB1546). Scale bar represents 5 μm.

**References**

Sieber, J.R., Sims, D.R., Han, C., Kim, E., Lykidis, A., Lapidus, A.L., *et al.* 2010. The genome of *Syntrophomonas wolfei*: New insights into syntrophic metabolism and biohydrogen production. *Environ Microbiol* 12: 2289–2301.
